# Degradation of Polylactic Acid Polymer and Biocomposites Exposed to Controlled Climatic Ageing: Mechanical and Thermal Properties and Structure

**DOI:** 10.3390/polym15142977

**Published:** 2023-07-08

**Authors:** Adam Vašíček, Petr Lenfeld, Luboš Běhálek

**Affiliations:** Faculty of Mechanical Engineering, Technical University of Liberec, Studentska 1402/2, 46117 Liberec, Czech Republic

**Keywords:** polylactic acid, biocomposite, buckwheat husks, egg shells, degradation, controlled climate ageing

## Abstract

This paper deals with the study of the degradation of polylactic acid (PLA) material structures and biocomposite systems with a PLA matrix containing ground natural particulate waste fillers, buckwheat husks and egg shells. Waste fillers were used without difficult cleaning operations to describe the effect of the raw waste material on PLA. Biocomposites with raw waste materials are increasingly coming to the forefront in car interiors and packaging products. The prepared material systems were exposed to controlled climatic ageing simulating long-term solar radiation and cyclic outdoor conditions. The degradation of the biocomposite systems was evaluated via thermal (differential scanning calorimetry) and mechanical properties (tensile and flexural tests, Charpy impact toughness). In addition to evaluating the degradation of the material structures using standardized tests, the influence and effect of controlled climatic ageing was visually assessed using SEM images (electron microscopy) of the surfaces and fracture surfaces of the test specimens.

## 1. Introduction

There is now a growing interest in environmental sustainability, and thus, in biopolymers to replace synthetic polymers that are not biodegradable and have a negative environmental impact [1,2]. Biopolymers are degraded into low molecular weight natural substances when exposed to environmental influences, microorganisms, elevated temperatures or ambient climates [3]. In 2022, 2.17 million tonnes of bioplastics were produced worldwide, with polylactic acid accounting for the largest production (20%) [4]. Polylactic acid (PLA) is considered a promising successor to commonly used synthetic polymers with similar properties to, for example, polyethylene terephthalate (PET) [5]. Among the primary advantages of PLA is precisely its biodegradability and the possible formation of both amorphous and semicrystalline phases, as the ratio of L,D-isomers affects the crystallinity or biodegradation rate of the polymer [5,6,7,8]. PLA has been applied in packaging materials such as fruit boxes, pouches or beverage cups [9]. PLA in copolymerization, e.g., with methacrylic acid as a cross-linked polymer, has been applied as a thickener for printed fabrics [10]. Studies have also looked at the branched PLA structure, which offers excellent processing and injection moulding properties [11]. The primary source for PLA production is starch-rich agricultural products such as corn or potatoes [12]. PLA production from corn stover or seaweed has also been investigated [13]. 

PLA changes its mechanical and optical properties due to thermal, oxidation or photodegradation processes during the product’s lifetime [14]. The marine ecosystem can lead to biopolymer degradation, manifested by swelling or hydrolysis [15]. Polymers undergo hydrolytic degradation due to moisture, as water molecules diffuse into the polymer and cause ester and primary chain cleavage. The degradation occurs mainly in the amorphous section; the crystalline section is more stable [16]. Exposure to solar UV radiation leads to bond breaking, i.e., ageing and possible degradation of PLA [17,18]. Under the influence of incident photons of UV radiation, polymer chains undergo cleavage or disintegration of the crystal structure of the polymer [16]. The effect of UV radiation can be simulated by outdoor exposure, where samples are placed in exposure racks and exposed to sunlight for 12–18 months, according to ASTM D5272-08 [19]. Currently, xenon test chambers are often used, where photodegradation is simulated with accelerations of 2:1, 5:1 and 63:1 [18].

In terms of PLA performance, fillers are added to the matrix. This creates a composite structure that can lead to better mechanical properties and lower product costs. In order to maintain the biodegradability of PLA, natural waste fillers are increasingly being used, which can be degraded by environmental conditions [20]. Natural fillers are either fibrous or particulate [21,22]. Among the fibrous ones, sisal, banana, bamboo, hemp and pineapple fibres are used [21]. Suitable particulate fillers are, e.g., buckwheat husks and onion skins. Buckwheat husks are a waste product of buckwheat production that cannot be used as livestock feed. They are used, for example, as fuel for boilers. Buckwheat husks have both antioxidant and bacteriostatic properties and are composed of organic and inorganic substances [22,23]. Egg shells have also been studied as particulate filler for PLA [24]. Egg shells are a widespread waste product of food, bakery and poultry processing plants [24,25]. Surveys in 2002 reported that egg shells can be used as fertilizers or as an ingredient in animal feed, and about one-third of egg shells end up in landfills [26]. They can also be applied as adsorbents for various unwanted organic and inorganic compounds from the waste segment [25].

## 2. Materials and Methods

**Figure 1:**
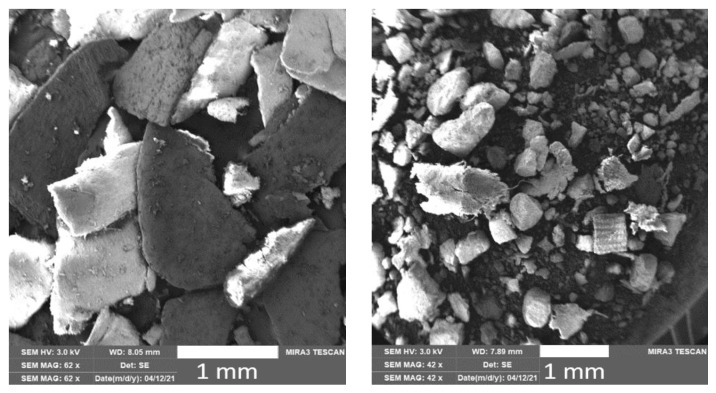
SEM images of ground buckwheat husks (**left**) and egg shells (**right**).

The PLA matrix used was commercially available PLA Ingeo 3001D [27], suitable for injection moulding, which was purchased (NatureWorks, Ingeo 3001D, Nakhon Sawan, Thailand). The PLA polymer contains more than 99% L-lactic acid and up to 1% D-lactic acid. The biocomposite materials were prepared from PLA biopolymer matrix and natural waste fillers, buckwheat husks and egg shells. Buckwheat husks (BH), which were free from impurities (dust and unwanted contaminants), and egg shells (ES) were dried and subsequently milled (Figure 1) using a 0.75 mm sieve on a mill (Retsch GmbH, Retsch SM 300, Haan, Germany).

### 2.1. Preparation of PLA Test Samples

The granulate of unfilled PLA matrix, ground egg shells and buckwheat husks were vacuum dried (Binder GmbH, VD53, Tuttlingen, Germany) for 24 h at 50 °C after moisture removal. The biocomposite materials were prepared by compounding on an extruder (Collin Lab & Pilot Solutions GmbH, Lab-Line ZK 25, Maitenbeth, Germany) with a granulator (Econ GmbH, Econ Ewa 10, Bergern, Austria) using the following process parameters: extruder temperature profile 135 to 175 °C (extrusion head), extruder screw speed 150 rpm and granulator knife head speed 3000 rpm. The compounding produced granular PLA biocomposites with the addition of 20 wt% ground buckwheat husks (PLA/BH) and egg shells (PLA/ES). The composition of the material structures and biocomposite systems and their designation are shown in Table 1. Type 1A test bodies according to ISO 527-2 were produced from unfilled PLA granulate and biocomposite granulate (Arburg GmbH + Co KG, Arburg Allrounder 320 C, Lossburg, Germany) via injection moulding. The injection parameters were the following: melt temperature, 190 °C; tempering medium temperature, 20 °C; batch volume, 36 cm^3^; injection pressure, 50 MPa; clamping force, 400 kN and total cycle time, 60 s. These parameters were evaluated as optimal for the production of the samples and were also based on the datasheet.

### 2.2. Ageing of Test Samples

The test bodies were subjected to controlled climatic ageing according to DIN 75 220 in a solar chamber (Vötch, SUN 3600, Balingen, Germany) with MH 2 × 4 kW radiators under two different controlled climatic ageing conditions. In the first case, the test bodies were loaded with a long-term test under constant irradiation conditions (enclosure temperature of 42 ± 3 °C, relative humidity of 65 ± 5%, irradiation intensity of 1000 ± 100 W·m^−2^) with a total irradiation time of 240 h. The aim of these conditions was to simulate long-term solar irradiation. The second controlled climatic ageing condition was a cyclic test (cyclical) that was conducted according to DIN 75 220, where the first 15 cycles simulated a dry climate, and then 10 cycles simulated a humid climate. Each cycle lasted 24 h, and each cycle had different climatic parameters (humidity, temperature and irradiation intensity) for the two climates. The total cycling time was 600 h. The set parameters of the cycling test simulated outdoor climatic conditions that last 4 years under standard natural effects.

### 2.3. Thermal Analysis (Differential Scanning Calorimetry)

The thermal properties of the biocomposites were evaluated using a calorimeter (Mettler Toledo, DSC 1/700, Greifensee, Switzerland) according to ISO 11357 standard for the evaluation of non-isothermal properties of samples. The measurements were performed on 6 ± 0.5 mg samples heated from 0 to 200 °C at a heating rate of 10 °C/min. After the 1st heating isotherm, the samples were heated to 200 °C for 3 min to remove thermal history. Subsequently, the temperature was reduced to an initial 0 °C, and the 2nd heating started. DSC analysis was performed in the presence of nitrogen at a flow rate of 50 mL/min. The enthalpy of secondary (cold) crystallization (ΔH_sc_), enthalpy of recrystallization (ΔH_rc_), enthalpy of crystallite melting (ΔH_m_), the temperature of secondary (cold) crystallization (T_sc_), the temperature of recrystallization (T_rc_) and the temperature of crystallite melting (T_m_) were recorded from the 1st heating step.

The degree of crystallization (χ_c_) was calculated according to Equation (1) as follows [28]:

χ_c_ = (ΔH_m_ − ΔH_rc_ − ΔH_sc_ − 100)/ΔH^0^_hm_  [%] (1)

where ΔH^0^_hm_ denotes the enthalpy of the melting of perfectly crystalline PLA (93 J/g).

### 2.4. Study of the Structure on Electron Microscope

The study of surfaces and fracture surfaces of unfilled PLA matrix and biocomposite structures was performed using a scanning electron microscope (SEM) (Tescan, TESCAN MIRA 3, Brno, Czech Republic). Samples were taken from the test bodies and attached to the targets using carbon tape. Before scanning, the individual samples were coated with a platinum/palladium metal mixture with a coating thickness of 6 nm, using an instrument (Leica, Leica EM ACE200, Munich, Germany) to ensure surface conductivity. The deposition was carried out via physical vapor deposition (PVD) in a protective atmosphere using argon. The test bodies for studying the fracture surface microscopy were prepared and fractured after using liquid nitrogen before breaking the sample. This does not affect the fracture surface as in the case of samples after, for example, tensile or Charpy impact toughness testing.

### 2.5. Mechanical Properties 

The test samples were conditioned according to ISO 291 at 35 °C with 62% relative humidity for 240 h before testing. The test pieces were then subjected to mechanical, tensile, bending and impact strength tests. These measurements were always taken on 10 test samples. The tensile properties were measured according to ISO 527 using an instrument (TIRA GmbH, TiraTest 2300, Schalkau, Germany) with a strain gauge (MF GmbH, MFL-300B, Velbert, Germany). The loading rate for the calculation of the tensile modulus was 1 mm/min until the failure of the body. The initial jaw spacing was 115 mm, with an initial gauge length of 50 mm and a preload of 2 N. The measured quantities were the tensile modulus (E_t_) and the ultimate tensile strength (σ_m_). The bending properties were measured according to ISO 178 using an instrument (Tinius Olsen, Hounsfield H10KT, Salfords, UK) with a sensing head of 500 N. The loading rate was 2 mm·min^−1^ at a preload of 2 N in three-point bending. The output of the measurements was the flexural modulus (E_f_) and the flexural strength (σ_fM_). The impact toughness of Charpy (a_cU_) according to ISO 179 was measured using an instrument (Instron, Resil Ceast S.p.A, Norwood, MA, USA). The body was broken by striking the narrower side of the test body without indentation.

## 3. Results and Discussion

### 3.1. Differential Scanning Calorimetry (DSC)

The unfilled PLA material sample reached a degree of crystallinity of 1.9% at a secondary crystallization peak of 115.9 °C (Table 2). The morphological structure of the sample after processing showed a highly disordered structure. The addition of 20 wt% of ground waste natural fillers resulted in a significant increase in the degree of crystallinity; for the PLA/ES biocomposite, the degree of crystallinity was 7.7% (an increase of about 4 times), and for the PLA/BH biocomposite, the degree of crystallinity was 12.4% (increase of about 6.5 times). The addition of waste fillers led to a higher degree of crystallinity, as observed, for example, by Koutsomitopoulou et al. [29] when olive pith powder was added to the PLA polymer. Similarly, Sivagnanamani et al. [24] measured a higher degree of crystallinity in the PLA by adding ground egg shells.

The biocomposite materials PLA/BH and PLA/ES were secondary crystallized at a lower temperature than the unfilled PLA. Adding natural filler to the biopolymer system leads to a higher crystal structure formation and lowers the temperature at which the PLA secondary crystallizes by about 10 °C. 

The exposure of the biocomposite samples to controlled climatic ageing significantly changed the degree of crystallinity, especially for the biocomposites filled with ground buckwheat husks (PLA/BH). After long-term (LA) and cyclic (CA) controlled ageing, the samples showed a higher degree of crystallinity by approximately four times than the PLA/BH composites that were not exposed to the climate chamber (higher degree of crystallinity was measured for long-term (LA) controlled climatic ageing). A similar increase (approx. four times) was also observed for the unfilled PLA polymer (higher degree of crystallinity was again measured for long-term (LA) controlled climatic ageing). In contrast, for the PLA/ES biocomposite, the effect of climate-controlled ageing on the degree of crystallinity was not statistically significant for long-term climate-controlled ageing, but a higher degree of crystallinity was measured for cyclic (CA) climate-controlled ageing, by approximately a 25% increase. 

### 3.2. Study of the Structure on Electron Microscope

**Figure 2:**
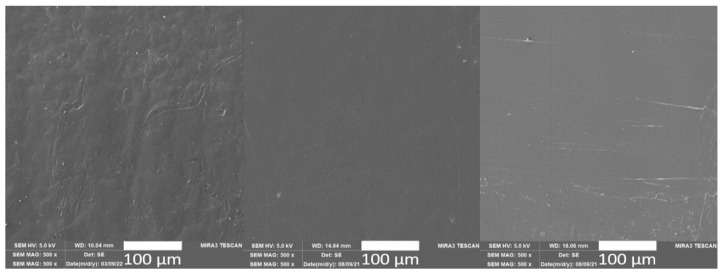
SEM images of the surface of unfilled PLA before exposure to controlled climatic ageing (**left**), after the effects of long-term climatic ageing (PLA/LA, 240 h, **middle**) and after the effects of cyclic controlled ageing (PLA/CA, 600 h, **right**).

The surface texture of the unfilled PLA and unfilled PLA samples exposed to long-term (PLA/LA) and cyclic (PLA/CA) climate-controlled ageing (see Figure 2) exhibits a smooth surface without significant damage or cracks. 

**Figure 3:**
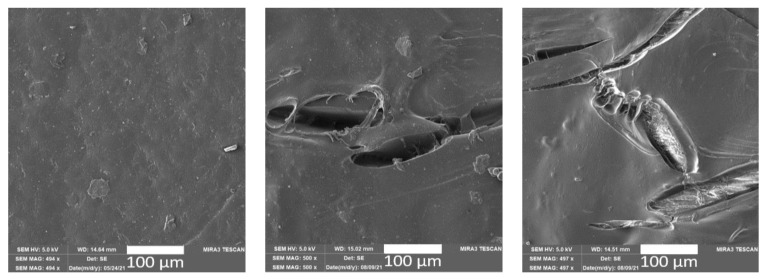
SEM images of the PLA/BH biocomposite surface before exposure to climate-controlled ageing (**left**), after the effects of long-term climate ageing (PLA/BH/LA, 240 h, **middle**) and after the effects of cyclic controlled ageing (PLA/BH/CA, 600 h, **right**).

**Figure 4:**
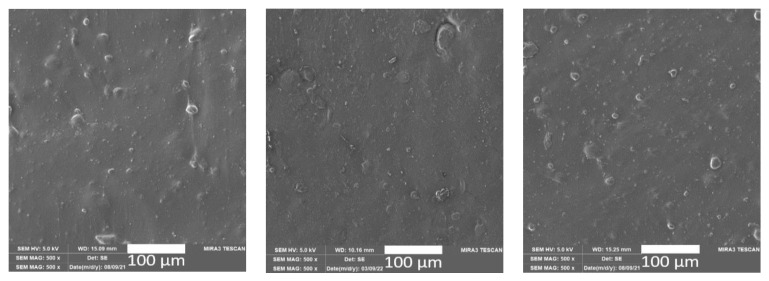
SEM images of the PLA/ES biocomposite surface before exposure to climate-controlled ageing (**left**), after the effects of long-term climate ageing (PLA/ES/LA, 240 h, **middle**) and after the effects of cyclic controlled ageing (PLA/ES/CA, 600 h, **right**).

The addition of ground natural waste fillers led to a visible effect on the surface of the PLA/BH and PLA/ES biocomposite structures (Figure 3 and Figure 4) in terms of the surface tribology. Both the buckwheat husks and egg shell particles are free of clumps and have good filler distribution and dispersion. 

After the effects of long-term and cyclic controlled ageing, the PLA/BH biocomposites show significant disruption of the surface structure, and cracks and fissures with visible fibrils on the surface. In contrast, the surface structures of the PLA/ES biocomposite do not show significant surface destruction after controlled climatic ageing (see Figure 3).

**Figure 5:**
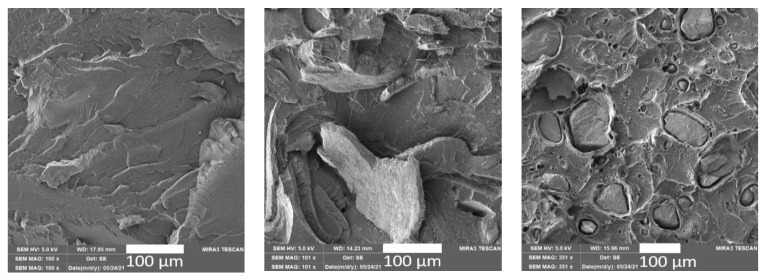
SEM images of fracture surfaces of unfilled PLA (**left**), PLA/BH biocomposite (**middle**) and PLA/ES (**right**) at the initial state.

The fractured structure of the unfilled PLA (Figure 5) exhibits a brittle type of failure. The buckwheat husks in the biopolymer system do not have 100% adhesion to the PLA matrix, and the size and properties of these particles can lead to structural defects and affect the mechanical properties. Jalbrzykowski et al. [22] similarly observed a lack of adhesion between the filler and matrix in the fracture of the biopolymer structure for the PLA polymer with the addition of buckwheat husks, which was probably due to the sensing of the test body after tensile testing. The egg shells in the PLA matrix do not have good adhesion, as seen in Figure 5. The waste filler may act as a structural defect. This can be eliminated according to Sivagnanamani et al. [24]. They removed the impurities and inner membranes of the egg shells before grinding the egg shells. After grinding, they achieved a particle size of fewer than 25 microns, and subsequently observed strong interfacial adhesion in the biopolymer system. Cleaning the egg shells before milling and actual application could lead to a more favourable particle adhesion to the PLA matrix.

### 3.3. Tensile Properties

The tensile modulus of the unfilled PLA polymer was measured to be 3644 MPa (Table 3). The addition of ground buckwheat husks to the PLA biopolymer matrix increased the tensile modulus by about 15%, and the ground egg shells increased the tensile modulus by about 20%. The exposure of the unfilled PLA and PLA/BH and the PLA/ES biocomposite samples to controlled climatic ageing decreased the tensile modulus. For the unfilled PLA, the effect of long-term climatic ageing (PLA/LA) led to a decrease of about 5% in the tensile modulus, and the effect of cyclic controlled ageing (PLA/CA) led to a decrease of about 12%. For the PLA/BH biocomposite, there was a similarly higher decrease in the tensile modulus for the cyclic controlled ageing parameters of about 11% (for PLA/BH/CA) and about 10% for the PLA/BH/LA biocomposite. In contrast, the PLA/ES biocomposite material had a higher decrease of about 12% in the modulus for long-term climate-controlled ageing (PLA/E/LA) and a decrease of about 5% for cyclic controlled ageing. 

The ultimate tensile strength of the unfilled PLA was measured to be 63.6 MPa, and an increase in this value (about 10%) was measured for the samples after long-term ageing (PLA/LA). After cyclic ageing, the values are almost identical. The addition of the natural raw waste materials BH and ES led to a decrease in the ultimate strength value of about 25%. The addition of filler led to the forming of a heterogeneous structure with limited adhesion at the interfacial interface between the matrix and filler for the biocomposites PLA/BH and PLA/ES. Exposing the biocomposite samples to controlled long-term and cyclic climatic ageing did not significantly affect the ultimate tensile strength, and the change in the values is statistically insignificant.

### 3.4. Impact Toughness

The impact toughness of Charpy was significantly reduced by the addition of ground waste fillers to the PLA biopolymer matrix (Table 4). While the unfilled PLA biopolymer had a Charpy impact toughness value of 18.6 kJ/m^2^, the PLA/BH biocomposite had a decrease of about 55%, and the PLA/ES biocomposite had a decrease of 40% due to structural defects at the interfacial interface of the particulate filler. Semicrystalline polymers or composites generally exhibit a coarser fracture structure, which is produced by crack propagation at the crystalline or matrix–filler interface. Exposing the unfilled PLA samples to controlled climatic ageing increased the Charpy impact toughness value by approximately 30% for both climate conditions. The increase was probably due to the higher degree of crystallinity after the effects of climate exposure. 

The increases in the Charpy impact toughness values for the samples that were exposed to controlled climatic ageing were measured not only for the unfilled PLA, but also for the PLA with the addition of buckwheat husks and egg shells to the biopolymer matrix. The increase in the impact toughness value was about 20% for the PLA/BH biocomposite after the effects of long-term and cyclic controlled ageing. For the PLA biocomposite samples containing egg shells, the Charpy impact toughness values increased by about 25% after the effects of controlled climatic ageing. Thus, when compared with the buckwheat husks, the Charpy impact toughness was higher for the ground egg shells, but lower than that for the unfilled PLA matrix.

### 3.5. Flexural Properties

In addition to the uniaxial tensile loading of the test bodies, a three-point bending test was performed on prepared specimens of the unfilled PLA and biocomposites PLA/BH and PLA/ES, which resulted in the multi-axial loading of the test body. 

The flexural modulus of the pure unfilled PLA was measured to be 3066 MPa (Table 5). The addition of ground buckwheat husks to the biopolymer matrix increased the flexural modulus by about 12%. In comparison, the addition of ground egg shells led to an increase of about 30% in the flexural modulus. Manshor et al. [30] achieved an increase in the flexural modulus for the PLA polymer samples by adding ground durian shells. Long-term ageing (PLA/LA) increased the flexural modulus value by about 10%, and cyclic ageing (PLA/CA) increased it by about 12%. Similar to the unfilled samples, the climate-controlled ageing of the PLA/BH samples increased the flexural modulus, with long-term ageing (PLA/BH/LA) resulting in an increase of 18%, and cyclic ageing (PLA/BH/CA) resulting in an increase of 13% in the flexural modulus. Thus, long-term climate-controlled ageing has a stronger effect than the unfilled PLA. The biopolymer composite systems containing egg shells (PLA/ES) showed an increase of about 8% in the flexural modulus after subjecting the samples to long-term ageing, and an increase of about 6% after cyclic ageing. The measured values show that controlled climatic ageing increases the flexural modulus of the biocomposite systems and unfilled PLA. Thus, in contrast to the tensile test, exposing the unfilled PLA and biocomposite PLA/BH and PLA/ES samples to controlled climatic ageing increased the flexural modulus.

The flexural strength of the unfilled PLA was measured to be 101.3 MPa. Controlled climatic ageing increased the flexural strength value for the PLA/LA and PLA/CA samples by 11%. After processing during their experiments, Ramesh et al. [31] achieved similar flexural strengths for the injection moulded PLA samples. The addition of raw waste materials (BH and EC) led to a decrease in the flexural strength of the biopolymer composite systems. The buckwheat husks decreased the flexural strength by 17%, and the ground egg shells decreased the flexural strength by 12%. The PLA/BH biocomposite samples subjected to long-term and cyclic controlled ageing achieved more or less similar flexural strength values, with long-term ageing increasing the value by about 5%. The test bodies with egg-shell-blended biocomposites (PLA/ES) exposed to controlled climatic ageing showed a similar trend to the buckwheat husks. Thus, the flexural strength values after controlled climatic ageing were almost identical to those measured for the samples without ageing.

## 4. Conclusions

In recent years, biopolymer materials and biopolymer composites have become one of the most emerging research areas. PLA-based materials and biocomposites with natural fillers have great potential for a wide range of applications and are beginning to gain significant importance. The use of biopolymers and biocomposites is no longer only for packaging, film and disposable products, but also for technical products in view of the increasing demands of industrial practice. Furthermore, the indisputable advantage in terms of application potential is not only the knowledge of the properties of biocomposites, but especially the knowledge of the effects of climate and controlled climatic ageing on the final and performance properties of biocomposites in terms of environmental sustainability.

The experimental results obtained from the evaluation of the degradation effects of controlled climatic ageing on the properties of the unfilled PLA matrix and PLA biocomposite systems containing buckwheat husks and egg shells were related to the matrix used, the type of filler and the parameters of long-term and cyclic controlled ageing. 

The degree of crystallinity was increased several times by adding natural waste fillers to the PLA matrix, confirming the results and conclusions known so far in this field as observed by Koutsomitopoulou et al. [29] and Sivagnanamani et al. [24]. At the same time, the degree of crystallinity increased significantly due to the effects of controlled climatic ageing for both the unfilled PLA matrix and the biocomposites filled with ground buckwheat husks. In contrast, for the PLA/ES biocomposite, the effect of controlled climatic ageing on the degree of crystallinity was statistically significant only for cyclic controlled climatic ageing.

When the mechanical properties were evaluated via tensile testing, it was found that adding ground buckwheat husks and egg shells to the PLA biopolymer matrix resulted in an increase of about 20% in the modulus compared to the unfilled PLA biopolymer. 

The exposure of the unfilled PLA and biocomposite PLA/BH and PLA/ES samples to long-term and cyclic controlled climatic ageing decreased the tensile modulus for all material systems and structures evaluated. 

The tensile strength of the unfilled PLA biopolymer increased after long-term climatic ageing. In contrast, cyclic ageing was statistically insignificant. The addition of natural raw waste materials, ground buckwheat husks and egg shells decreased the ultimate strength value by about 25%. Exposing the prepared biocomposite structure samples to controlled long-term and cyclic climatic ageing did not significantly affect the tensile strength, and the change in the values was statistically insignificant. 

The flexural modulus increased significantly with the addition of ground waste fillers to the polymer matrix, as observed by Manshor et al. [30], especially after adding ground egg shells to the matrix. For the buckwheat husks, the increase was about half of that of the egg shells. The results confirm the findings of other publications and studies. The application of controlled climatic ageing increased the flexural modulus for both the unfilled PLA matrix and the biocomposite structures. The highest increase was measured when buckwheat husks were used. Long-term (LA) controlled climatic ageing has a higher effect on the flexural modulus for the biocomposite structures. In contrast to the tensile test, exposing the samples to controlled climatic ageing increased the flexural modulus.

The PLA matrix specimens subjected to controlled climatic ageing showed an increase in the flexural strength value, confirming the known findings from available publications. The addition of raw waste materials (BH and ES) led to a decrease in the flexural strength of the biopolymer composite systems by about 15% on average. Controlled climatic ageing has almost no effect on the flexural strength values of the biocomposites filled with ground buckwheat husks and egg shells. 

The impact toughness value of Charpy decreased significantly by adding ground waste fillers to the PLA biopolymer matrix. Exposing the unfilled PLA samples to controlled climatic ageing increased the Charpy impact toughness value by approximately 30% for both climate conditions. The increase in the Charpy impact toughness values for the samples exposed to controlled climatic ageing was measured for both the unfilled PLA and the biopolymer matrix with the addition of both buckwheat husks and egg shells.

The surface texture of the unfilled PLA samples before and after the effects of controlled climatic ageing showed a smooth surface without significant damage and cracks. The addition of ground natural waste fillers resulted in a visible effect on the tribology of the surface of the biocomposite structures. However, the particles of the added waste materials have good distribution and dispersion in the biopolymer matrix. The PLA/BH biocomposites show significant surface structure distortion after the effects of long-term and cyclic controlled ageing. In contrast, the surface structures of the PLA/ES biocomposites do not show significant surface destruction after controlled climatic ageing. The fracture structure of the unfilled PLA shows a brittle type of failure. The ground egg shells and buckwheat hull particles in the biopolymer matrix did not show good adhesion, which could be because since they were not chemically cleaned, as confirmed by other studies and publications such as that by Sivagnanamani et al. [24].

The knowledge gained about biopolymers and biopolymer composites, including about the characterization of their properties after the effects of degradative climate-controlled ageing, is important in terms of potential applications and environmental and ecological aspects.

## Figures and Tables

**Table 1:** Sample compositions.

Sample Marking	Matrix	Filler	Controlled Climatic Ageing
PLA	PLA	-	-
PLA/LA	PLA	-	Long-term
PLA/CA	PLA	-	Cyclical
PLA/BH	PLA	20 wt% BH	-
PLA/BH/LA	PLA	20 wt% BH	Long-term
PLA/BH/CA	PLA	20 wt% BH	Cyclical
PLA/ES	PLA	20 wt% ES	-
PLA/ES/LA	PLA	20 wt% ES	Long-term
PLA/ES/CA	PLA	20 wt% ES	Cyclical

**Table 2:** Thermal analysis (DSC) data of PLA biocomposites exposed to long-term and cyclic controlled climatic ageing. Measured values are from the first heating. The symbols are explained as follows: the enthalpy of secondary (cold) crystallization (ΔH_sc_); enthalpy of recrystallization (ΔH_rc_); enthalpy of crystallite melting (ΔH_m_); the temperature of secondary (cold) crystallization (T_sc_); the temperature of recrystallization (T_rc_); the temperature of crystallite melting (T_m_); degree of crystallization (χ_c_).

Material	ΔH_sc_ (J/g)	ΔH_rc_ (J/g)	ΔH_m_ (J/g)	Χ_c_ (%)	T_sc_ (°C)	T_rc_ (°C)	T_m_ (°C)
PLA	35.71	-	37.52	1.9	115.9	-	170.1
PLA/LA	30.06	0.48	37.33	7.3	103.3	155.7	168.6
PLA/CA	31.76	-	37.23	5.9	106.7	-	169.2
PLA/BH	20.95	0.64	30.78	12.4	104.5	155.7	169.0
PLA/BH/LA	-	1.60	38.67	49.8	-	152.7	168.3
PLA/BH/CA	-	1.90	36.15	46.0	-	150.1	167.7
PLA/ES	25.27	-	31.02	7.7	108.2	-	169.2
PLA/ES/LA	25.01	2.34	33.78	8.6	97.7	153.4	168.3
PLA/ES/CA	24.10	2.30	33.48	9.5	97.5	153.4	168.2

**Table 3:** Tensile properties of PLA biocomposites exposed to long-term and cyclic controlled climatic ageing.

Material	E_t_ (MPa)	σ_m_ (MPa)
PLA	3644 ± 32	63.6 ± 0.5
PLA/LA	3461 ± 154	70.0 ± 1.3
PLA/CA	3200 ± 75	64.2 ± 2.9
PLA/BH	4163 ± 29	47.8 ± 0.5
PLA/BH/LA	3727 ± 124	49.0 ± 2.0
PLA/BH/CA	3704 ± 554	47.4 ± 1.8
PLA/ES	4336 ± 58	48.9 ± 0.4
PLA/ES/LA	3798 ± 350	49.7 ± 0.8
PLA/ES/CA	4174 ± 159	47.7 ± 0.9

**Table 4:** Charpy impact toughness of PLA biocomposites exposed to long-term and cyclic controlled climatic ageing.

Material	a_cU_ (k/m^2^)
PLA	18.6 ± 1.5
PLA/LA	24.1 ± 1.1
PLA/CA	23.8 ± 1.0
PLA/BH	8.4 ± 1.5
PLA/BH/LA	9.8 ± 0.8
PLA/BH/CA	10.4 ± 1.0
PLA/ES	11.1 ± 1.4
PLA/ES/LA	14.0 ± 0.4
PLA/ES/CA	13.2 ± 1.6

**Table 5:** Flexural properties of PLA biocomposites exposed to long-term and cyclic controlled climatic ageing.

Material	E_f_ (MPa)	σ_fM_ (MPa)
PLA	3066 ± 77	101.3 ± 0.9
PLA/LA	3367 ± 45	112.7 ± 0.7
PLA/CA	3443 ± 79	112.5 ± 1.0
PLA/BH	3448 ± 171	84.5 ± 3.0
PLA/BH/LA	4084 ± 58	89.0 ± 7.7
PLA/BH/CA	3895 ± 79	84.3 ± 8.5
PLA/ES	3929 ± 56	89.2 ± 0.4
PLA/ES/LA	4266 ± 182	92.4 ± 2.2
PLA/ES/CA	4158 ± 213	89.1 ± 4.9

## Data Availability

The data presented in this study are available on request from the corresponding author.
